# Validating the rigour of adaptive methods of economic evaluation

**DOI:** 10.1136/bmjgh-2023-012277

**Published:** 2023-09-26

**Authors:** Akashdeep Singh Chauhan, Deepshikha Sharma, Abha Mehndiratta, Nidhi Gupta, Basant Garg, Amneet P Kumar, Shankar Prinja

**Affiliations:** 1 Department of Community Medicine and School of Public Health, Post Graduate Institute of Medical Education and Research, Chandigarh, India; 2 Center for Global Development, Washington, Columbia, USA; 3 Department of Radiation Oncology, Government Medical College and Hospital, Chandigarh, India; 4 National Health Authority, Ayushman Bharat PM-JAY, Government of India, New Delhi, India; 5 Department of Women and Child Development, Government of Haryana, Panchkula, Haryana, India

**Keywords:** health economics, health policy, cancer

## Abstract

**Background:**

There has been a lot of debate on how to ‘generalise’ or ‘translate’ findings of economic evaluation (EE) or health technology assessment (HTA) to other country contexts. Researchers have used various adaptive HTA (aHTA) methods like model-adaptation, price-benchmarking, scorecard-approach, etc., for transferring evidence from one country to other. This study was undertaken to assess the degree of accuracy in results generated from aHTA approaches specifically for EE.

**Methods:**

By applying selected aHTA approaches, we adapted findings of globally published EE to Indian context. The first-step required identifying two interventions for which Indian EE (referred to as the ‘Indian reference study’) has been conducted. The next-step involved identification of globally published EE. The third-step required undertaking quality and transferability check. In the fourth step, outcomes of EE meeting transferability standards, were adapted using selected aHTA approaches. Lastly, adapted results were compared with findings of the Indian reference study.

**Results:**

The adapted cost estimates varied considerably, while adapted quality-adjusted life-years did not differ much, when matched with the Indian reference study. For intervention I (trastuzumab), adapted absolute costs were 11 and 6 times higher than the costs reported in the Indian reference study for control and intervention arms, respectively. Likewise, adapted incremental cost and incremental cost-effectiveness ratio (ICER) were around 3.5–8 times higher than the values reported in the Indian reference study. For intervention II (intensity-modulated radiation therapy), adapted absolute cost was 35% and 12% lower for the comparator and intervention arms, respectively, than the values reported in the Indian reference study. The mean incremental cost and ICER were 2.5 times and 1.5 times higher, respectively, than the Indian reference study values.

**Conclusion:**

We conclude that findings from aHTA methods should be interpreted with caution. There is a need to develop more robust aHTA approaches for cost adjustment. aHTA may be used for ‘topic prioritisation’ within the overall HTA process, whereby interventions which are highly cost-ineffective, can be directly ruled out, thus saving time and resources for conducting full HTA for interventions that are not well studied or where evidence is inconclusive.

**WHAT IS ALREADY KNOWN ON THIS TOPIC**

- Adaptive or pragmatic health technology assessment (aHTA) methods are increasingly being used for evidence generation in situations where evidence from the ‘gold standard’ of locally conducted HTA is not available. Previous studies have reported on the different approaches of aHTA that can be used for adapting or transferring international evidence to local contexts. There has also been a lot of work around the development of different checklists, summarising the list of various factors that could potentially lead to uncertainties while generalising, transferring or adapting the results to other settings. However, there is no study that has measured the degree of accuracy in the findings generated from adaptive approaches for economic evaluation (EE) as compared with a traditional full EE.

**WHAT THIS STUDY ADDS**

- The present analysis is first of its kind to use selected aHTA approaches for EE (including literature review, quality and transferability appraisal, and costs, outcome, and price adjustments), and to assess it against the traditional full EE for measuring the validity and accuracy of the findings. It was observed that in terms of direction and magnitude, the adapted cost estimates varied considerably, while the adapted health outcome in the form quality-adjusted life-years did not differ much, when matched with the results of the traditional full EE. Finally, it is concluded that the findings derived from aHTA approaches should be interpreted with caution, even if the adapted results are generated using HTA evidence from a nation with similar characteristics to the decision country.

**HOW THIS STUDY MIGHT AFFECT RESEARCH, PRACTICE OR POLICY**

- The study has important policy implications and suggests that the aHTA approaches still cannot be considered as a replacement of the traditional HTA methods, even for decisions that require immediate evidence. However, aHTA as an approach may be considered as one of the tools in the overall traditional HTA process. First, aHTA may be used for summarising the existing evidence on the clinical efficacy, safety, cost and cost-effectiveness from the globally published EE or HTA reports. Second, aHTA can be used for ‘topic prioritisation’ in the schematic flow of HTA process, whereby those interventions which are highly cost-ineffective, can directly be ruled out, thus saving time and resources for conducting a full HTA for interventions that are not well studied internationally or where HTA evidence is inconclusive.

## Introduction

 In various low-income and middle-income countries (LMICs), there has been an increase in the uptake of evidence generated from health technology assessment (HTA) to better inform healthcare priority setting.^1^,^3^ An HTA assesses the effectiveness, safety and cost-effectiveness of new technologies and aids in various policy level decisions related to inclusion/exclusion of a technology under a health benefit package (HBP) and its reimbursement rates.^4 5^ Various developing economies have also institutionalised systems for generation of HTA evidence by creating governance structures and guidelines for appraising a wide range of technologies ranging from drugs, devices, diagnostics and healthcare programmes.^6^,^9^

An economic evaluation (EE), whether conducted alongside a randomised controlled trial or as a model-based analysis, is an integral component of the broader HTA report. It is a rigorous and time-consuming systematic process which requires intensive data needs and technical expertise.^4 10^ Ideally, all countries need to conduct their own HTA analysis for a specific technology by considering local evidence on cost, effectiveness, epidemiology, and other population and health system characteristics. However, time constraints, inadequate data and limited capacity often impedes the timely conduct of these HTA studies (or EE), especially in LMICs.^2^ This puts a lot of pressure on HTA agencies for the timely conduct of HTA studies, especially in situations where immediate evidence is required. For example, in India, there is a rapidly growing demand for HTA evidence from government health departments which receive continuous requests from pharmaceutical and medical device agencies as well as patient advocacy groups for timely decisions on including a particular technology/service in the HBP. In these circumstances, decisions could either be based on individual judgement or some form of rapid evidence or based on a full HTA report. However, with limited capacity and resources for doing a full HTA, adaptive methods could be the next best option for decision making. At times, when the evidence from the ‘gold standard’ of locally conducted HTA is not immediately available, there have been instances where researchers have used certain adaptive or pragmatic HTA (aHTA) methods to speed up the process of evidence generation.^11^ An aHTA has been defined as ‘a blanket approach for HTA methods and processes which are fit-for-purpose and focused on context-specific practicality considerations’.^11^

Broadly, the aHTA methods ‘leverage, adapt or transfer’ international evidence from published literature on EE or HTA reports to generate context-specific and locally relevant evidence, while accounting for uncertainties around transferability.^12^,^19^ There are a wide range of aHTA approaches which differ in scope, complexity and data requirements. Importantly, all these are not mutually exclusive approaches and have been used in many different forms. The benefit of aHTA approaches is that these are both less time-consuming, and pose minimum data requirements. Furthermore, it allows resources to be used for undertaking full HTA for interventions where international data is scanty or inconclusive.^11^ Dependency of aHTA on international literature is however its biggest limitation^11^ along with evidence being leveraged from countries, which are often high-income or middle-income that have different health system and population characteristics. Further, there could be variation in the methodological conduct of EE across nations. These differences could lead to uncertainty in the findings generated from an aHTA approach.

In the recent years, there have been a series of publications addressing the issue of geographic transferability of EE or HTA results.^20^,^24^ Globally, researchers have used or developed adaptive methods for transferring the results of an EE to the other country’s contexts. There has been a lot of work around the development of different checklists for assessing transferability. Furthermore, most of these studies focused on identifying potential factors that could cause uncertainties, while transferring or adapting results from one country setting to others.^20^,^25^ However, there has been no evidence that has measured the degree of accuracy in the findings generated from aHTA approaches. We undertook the present study to validate the findings generated from selected adaptive methods for EE. Validation was assessed by measuring the degree of accuracy or precisely how closely the adapted results matched with the findings of a full EE.

## Methods

**Figure 1:**
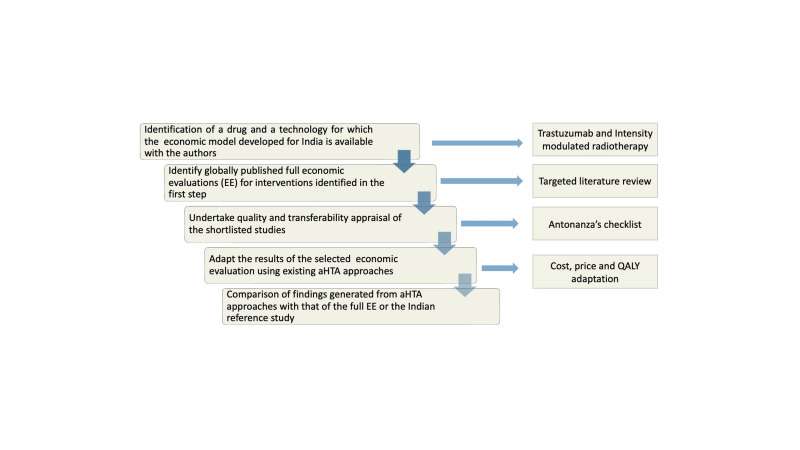
Methodological framework. aHTA, adaptive health technology assessment; QALY, quality-adjusted life-year.

The methodological framework is broadly described in figure 1. The first step required identifying two specific interventions (and disease areas) which had been evaluated for their cost-effectiveness in the Indian context and their economic models were available with the authors. The next step involved the identification of globally published EE (or HTA reports) for the selected interventions (identified in the first step) through a targeted literature review. The third step required undertaking an assessment of quality and transferability of the studies identified through the targeted literature review. In the fourth step, the outcomes of those EE studies which met appropriate transferability standards were adapted for Indian settings using selected aHTA approaches. Lastly, the adapted results were compared with the findings of the Indian EE (hereafter referred to as the ‘Indian reference study’).

### Identification of interventions

We selected a drug and a medical device for the present analysis. There are methodological differences in costing, valuation of consequences, as well as other structural assumptions such as time horizon, perspective, etc. when undertaking an EE for different types of healthcare technologies including drugs and medical devices.^26^ Therefore, we considered it relevant to undertake validation for both the two broad types of healthcare technologies seprately and assess any difference in the conclusion of aHTA, when conducted for a drug and a device.

We selected an anticancer drug, that is, trastuzumab and a radiation therapy technology—intensity-modulated radiation therapy (IMRT). Trastuzumab is indicated for management of various cancers, including breast cancer, oesophageal cancer and stomach cancer.^27^ Similarly, IMRT is also used for irradiating breast cancer, cervical cancer, head and neck cancer (HNC), prostate cancer, etc.^28^ Considering the scope of the available corresponding full EEs, we narrowed the focus on breast cancer and HNC as the disease area for the use of trastuzumab and IMRT, respectively.

Another reason for choosing these technologies and disease areas for validation was the fact that aHTA has gained prominence for use for developing standard treatment guidelines and reimbursement rates in the field of oncology.^29 30^ Furthermore, experience of India’s publicly financed insurance scheme— A*yushman Bharat Pradhan Mantri Jan Arogya Yojana (*PM-JAY) shows that the number of new technologies (drugs or devices) submitted for inclusion in the national HBP has been highest for cancer care. Moreover, among cancers, both breast and HNC are the most prevalent cancers globally and are associated with high treatment costs.^31 32^ Finally, since the authors had full access to the economic models of these published Indian cost-effectiveness studies, that is, trastuzumab and IMRT for breast and HNC, respectively, these were considered for the present analysis.^30 33^

### Identifying globally published EEs

A targeted literature review was undertaken for identifying globally published full EE (or HTA reports) on the cost-effectiveness of using adjuvant annual trastuzumab for treatment of breast cancer and IMRT for management of various HNC. A comprehensive search was undertaken in PubMed. Only peer-reviewed articles and HTA reports were searched. Abstracts, conference papers, reviews, opinions or commentaries were excluded. Search strategy was developed using specific keywords pertaining to technology, disease area, cost, cost-effectiveness, HTA, health outcome, etc. The key words were checked for controlled vocabulary under Medical Subject Headings of PubMed. The search was conducted in the month of May 2023 and was restricted to identification of only human studies published in the last ten years. A study was included if it matched the population, intervention, comparator and outcome (PICO) criteria of the Indian reference studies. For trastuzumab PICO criteria was defined as— P: human epidermal growth factor receptor 2 (HER2) positive breast cancer patients, I: adjuvant annual trastuzumab therapy, C: standard chemotherapy without trastuzumab, O: incremental cost per quality-adjusted life-year (QALY) gained. Similarly, for IMRT, PICO was defined as—P: HNC patients, I: IMRT, C: 3D-CRT, O: incremental cost per QALY gained. The detailed search strategy is provided in online supplemental appendix 1 and 2.

**Figure 2:**
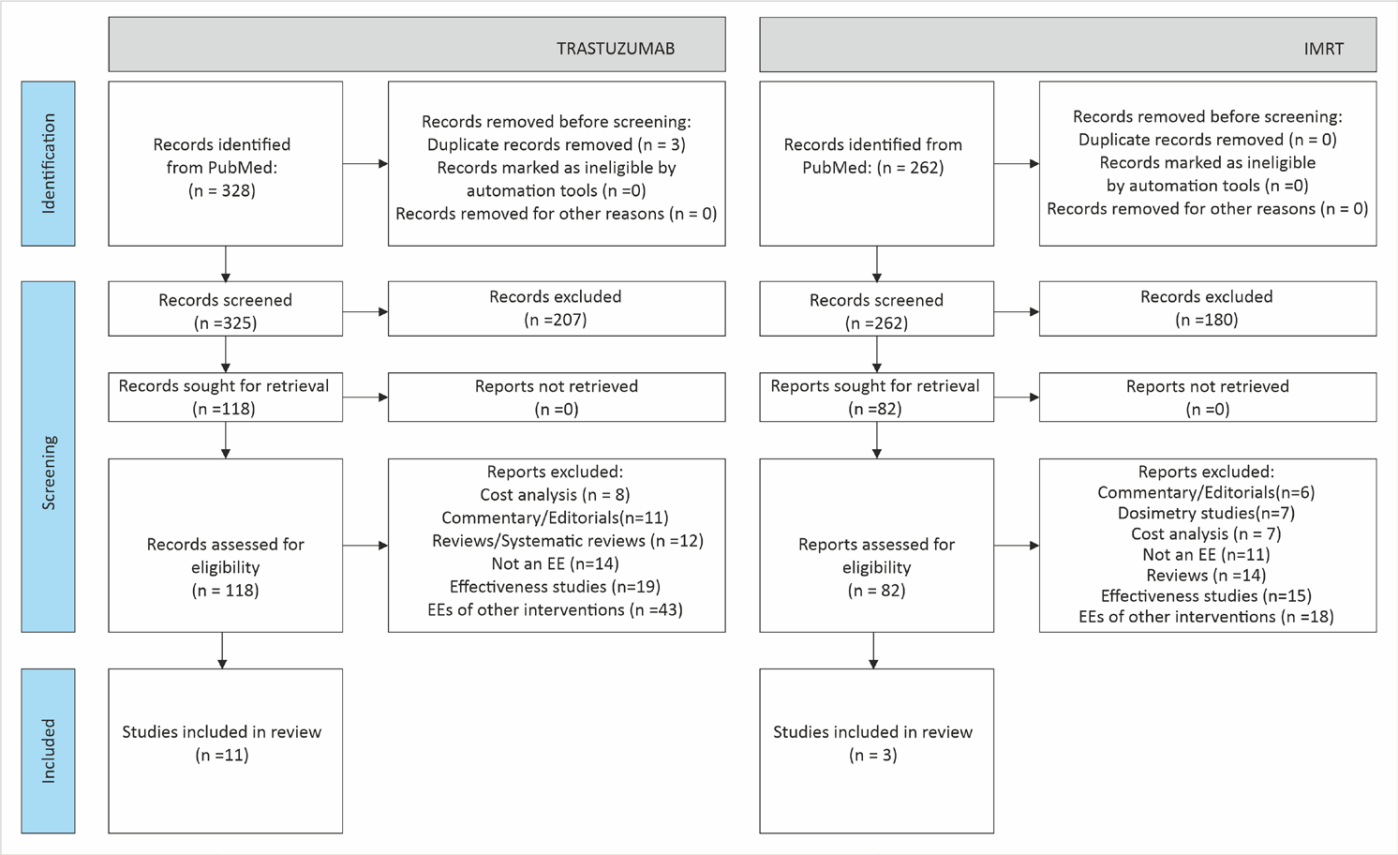
Preferred Reporting Items for Systematic Reviews and Meta-Analyses (PRISMA) diagram showing selection of studies. EE, economic evaluation; IMRT, intensity-modulated radiation therapy.

The studies were identified following the screening process as outlined in the PRISMA guidelines (figure 2). First, all the records retrieved from the search were screened by the title. The abstract of those records with potentially relevant titles were further screened for relevant outcomes in the form of costs, QALYs, incremental cost-effectiveness ratios (ICERs), etc. Lastly, papers identified through screening of titles and abstracts were further examined for eligibility by reviewing their full texts. Finally, all those studies that were full scale EE (or HTA reports) comparing two or more interventions (as per eligibility criteria mentioned above) were included. At this stage, a bibliographic search of the selected studies was also carried out to identify additional relevant articles. Two reviewers (ASC and DS) independently undertook the screening process, and any discrepancy was resolved in discussion with the third reviewer (SP). From each of the selected studies, data on country settings, study population, intervention, comparator, methodological characteristics (perspective, time horizon and discount-rate), cost (currency and price data), and health outcomes (life year and QALY) were extracted.

### Appraisal for quality and transferability

Assessment of quality and transferability of the selected EE was done using the Antonanza’s checklist (online supplemental appendix 3).^24^ Quality check was undertaken to assess the degree to which the selected studies followed best practices and standards of conducting an EE.^21 34^ Transferability was defined as ‘capacity to use the results of an EE in a setting different from the original one in which the technology was assessed’.^24^ Two reviewers (ASC and DS) independently screened the studies following the Antonanza’s checklist, any disagreement was settled in discussion with the third reviewer (SP). The Antonanza’s checklist was used because it gives an objective numerical score to an EE, which makes assessment easier based on a quantitative scale. Furthermore, a threshold score can also be set, and those EEs receiving a score below the threshold can directly be considered unsuitable for transferability.

This numerical score, also referred to as global transferability index (IT), is based on a mean of two partial indices—general IT_1_ and specific IT_2_.^24^ The general IT uses seven critical and sixteen non-critical objective factors. If a study does not pass through two or more critical objective factors, it receives a score of zero, and was knocked-out or considered non-transferable. The non-critical factors did not have any knock-out criteria, but had a relative weight based on their relevance for transferability.

The specific IT assesses the ‘level of difficulty that exists in applying or adopting the information in original study to new setting’.^24^ It is calculated based on four critical and eight non-critical subjective factors. The study was deemed non-transferable, if it did not clear any one of the critical subjective factors. Non-critical subjective factors were valued on a likert scale from 0 to 4.

The value of the global IT varies between 0 and 1, where 0 and 1 indicate lowest and highest value given to an EE based on its quality and transferable capacity. We set a thumb rule, in addition to knock-out criteria and included only those studies for adaptation that received a global IT of 0.5 and above.

### Adaptation of results to Indian settings

Due to variation in health system characteristics such as level of resource utilisation, resource valuation, payment mechanisms, healthcare delivery structure, efficiency, etc., it is difficult to generalise the cost estimated in an EE to other countries.^21 22^ Similarly, due to differences in population characteristics such as life expectancy, health status preference, casemix, disease spread, etc., it becomes challenging to generalise health outcomes to other countries.^21 22^ All these factors create a need for adapting the results of an EE.

Various aHTA methods such as rapid reviews, price-benchmarking, complete model adaptation, adaptation of model outcomes, adaption of international data-sets, etc., have been developed.^12^,^18^ As we did not have access to the original model underlying selected EE of other countries, we validated aHTA findings generated through literature review, adaptation of model outcomes and price-benchmarking. Moreover, these are also the more commonly performed approaches.

**Figure 3:**
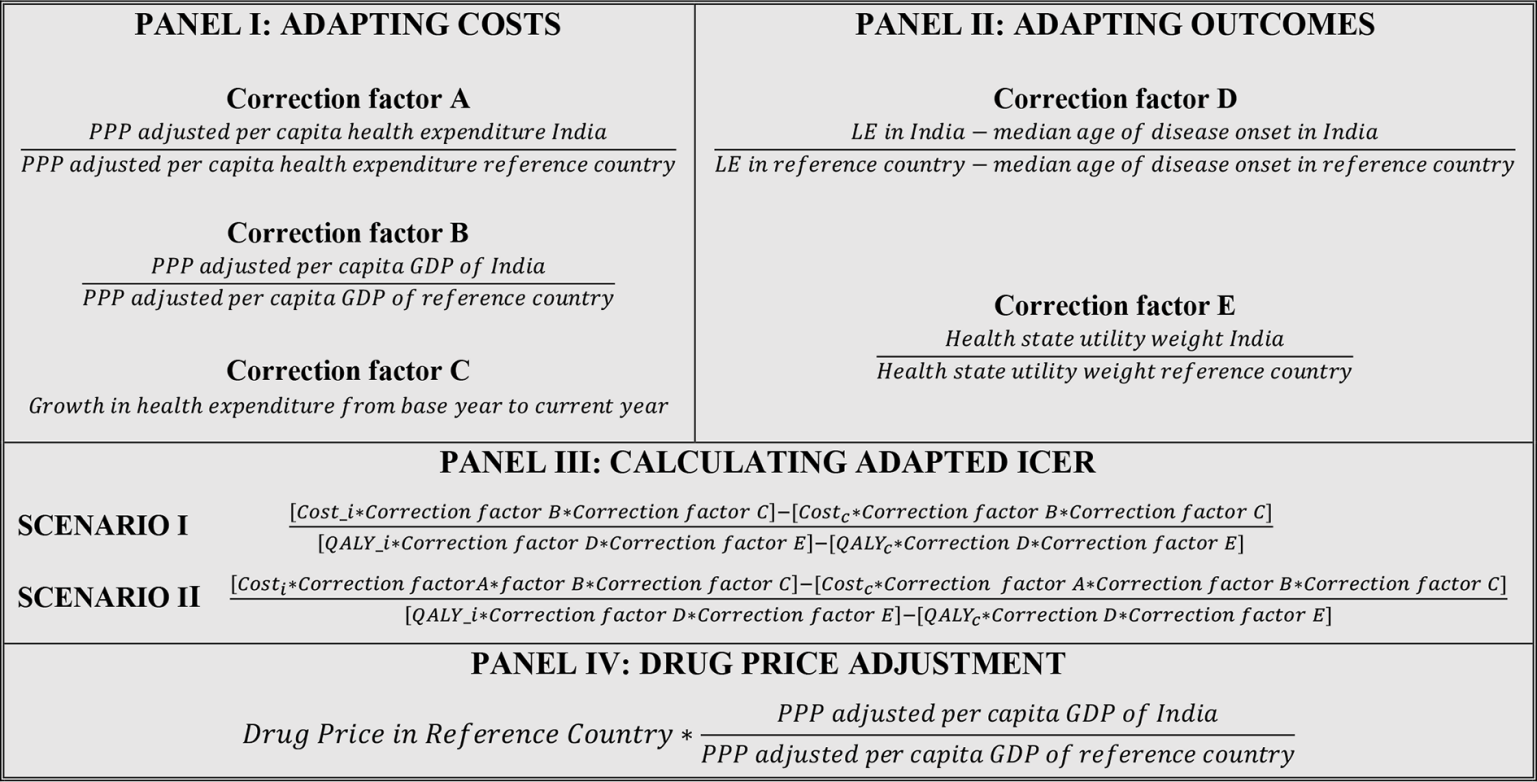
Adapting cost and health outcomes. c, comparator; GDP, gross domestic product; i, intervention; ICER, Incremental cost-effectiveness ratio; LE, life expectancy; PPP, purchasing power parity.

As a first step, we collated results of selected EEs, and directly compared it with findings and conclusion of the Indian reference study. Second, we applied three correction factors for adapting the cost reported in the selected EEs to the Indian context.^20^ These correction factors focused on fixing differences in level of resource utilisation, prices of healthcare services, and changes in prices over time (panel I of figure 3). The type and quantity of resource use were corrected by applying correction factor A, that is, ratio of per-capita health expenditure (purchasing power parity (PPP) adjusted) in India to per-capita health expenditure (PPP adjusted) of other countries, where the selected EE was conducted. For adjusting the prices of healthcare services, we used correction factor B, ratio of PPP adjusted per-capita gross domestic product of India (GDP) to the PPP adjusted per-capita GDP of other countries. To adjust difference in change in price over time, we applied inflation rates, that is, GDP deflators, as correction factor C. It was recognised that there could be over-adjustment of costs while using both correction factors A and B, as even correction factor B was sufficient to adjust for difference in the level and prices of resources. Hence, we conducted two distinct sets of analyses. In scenario I, we used only correction factors B and C to adjust costs. In scenario II, we used all the three corrections factors (A, B and C) for adjusting costs (panel III figures3  4). Each of the cost correction factors between India and the countries of selected EEs were calculated separately and have been reported in online supplemental file 1. The original cost outcome as reported in the selected EEs were first adapted by multiplying it with correction factor B (or both A and B). The resultant adapted estimate was then converted to Indian rupees (₹), using currency conversion rates, of the year in which the original selected EE was conducted. Lastly, the converted ₹ value was inflated (or deflated) to the year, by using correction factor C, in which the Indian reference study was conducted.

**Figure 4:**
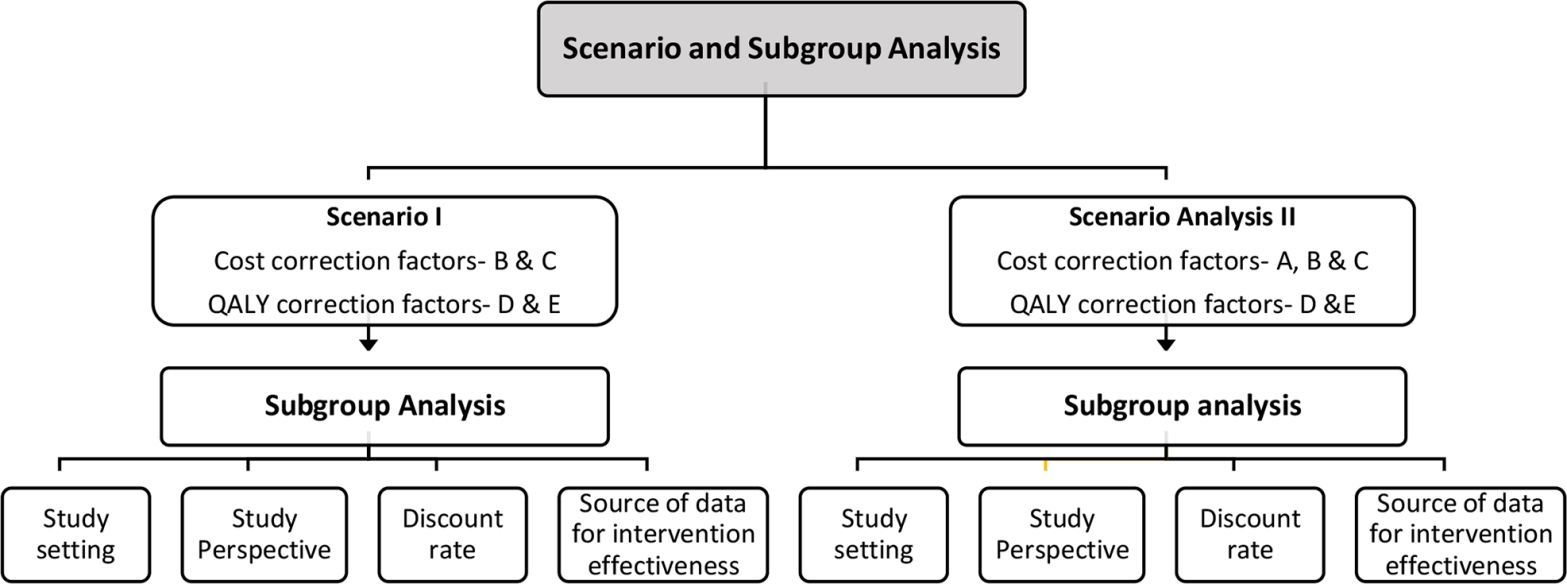
Scenario and subgroup analysis. QALY, quality-adjusted life-year.

We could not find any specific approach that has been used for adjustment of health outcome—QALY. As QALY is a combination of quantity and quality of life lived, we constructed two correction factors for adjusting for the difference in life expectancy and health status preference (panel II of figure 3). To adjust the former, correction factor D—ratio of difference of life expectancy and median age of onset of disease in India to the difference of life expectancy and median age of onset of disease in the other countries was used. For the latter, we used correction factor E—ratio of utility value of a similar health state (or states) used in the Markov model of the Indian reference study to the utility values mentioned in the selected EEs of other countries. The correction factors D and E were calculated separately for each of the selected EEs of other countries and are reported in online supplemental appendix 5.

Lastly, price-benchmarking was used to calculate a cost-effective price of trastuzumab for India, by multiplying the price of trastuzumab reported in the selected EE, by the ratio of PPP-adjusted per-capita GDP of India to the PPP- adjusted per-capita GDP of the other countries (panel IV of figure 3).

### Comparison of aHTA findings with the Indian reference study

The adapted cost and QALYs were subsequently used to calculate incremental outcomes including ICER (panel III of figure 3). These adapted findings were then compared with the results of the Indian reference study. Similarly, the cost-effective price estimated through the benchmarking analysis was compared with the price, at which trastuzumab was considered cost-effective in the Indian reference study.^30^ The technology was considered to be cost-effective, if the value of adapted ICER falls below the one-time GDP per-capita of India, as recommended in the guidelines of HTA Board of India.

#### Subgroup analysis

For both scenarios I and II, a subgroup analysis was also undertaken to identify potential factors that could contribute to uncertainties in the adapted findings (figure 4). These factors included study perspective (health system or societal), discount rate and study settings (high income or middle income). Additionally, we explored the possibility of the source of data for intervention effectiveness being a contributing factor to variations in the adapted findings. Therefore, we also performed a subgroup analysis to compare the adapted ICERs particularly for those studies that used the same effectiveness estimates as the Indian reference study, that is, from the HERA trial.

#### Patient and public involvement

Patients or the public were not involved in the design, conduct or reporting of this research study.

## Results

### Summary of included studies

#### Trastuzumab

A total of 11 EEs were identified for trastuzumab (table 1).^35^,^45^ Of these, seven were undertaken in middle-income countries,^35 37 38 40 42 43 45^ three were from high-income countries,^36 39 41^ while the remaining one study was a multicentre evaluation conducted for seven Latin-American countries.^44^ The Indonesian study was excluded from the adaptation exercise as it did not pass through two of the critical objective criteria, and hence received a transferability score of 0.^45^ Most of the studies had either used a healthcare system or payer perspective, while the EE from Thailand used a societal perspective.^40^ The Philippines’ study had undertaken analysis both from a societal as well as a healthcare system perspective.^35^ All the studies had used a lifetime horizon and undertaken discounting using a common rate of 3% or 3.5% or 5% for both cost and health outcomes. The Dutch EE undertook differential discounting using a rate of 4% for costs and 1.5% for health outcomes.^36^ The Indian reference study used a societal perspective, lifetime horizon and a common discount rate of 3%.^30^

**Table 1:** Summary of included studies for trastuzumab

Country, year	Perspective	Time horizon	Discount rate	Conclusion	Global transferability index
Philippines 2019^35^	Healthcare system and societal	Lifetime	3.5%	Not CE	0.70
Netherlands 2017^36^	Dutch healthcare system	Lifetime	4% costs;1.5% outcomes	CE at 1-time GDP per capita	0.55
Brazil 2022^37^	Brazilian public health system	Lifetime	5%	CE at 3-time GDP per capita	0.60
Iran 2014^38^	Healthcare system	Lifetime	3.5%	Not CE	0.55
Cyprus 2020^39^	Cyprus NHS payer	Lifetime	3%	CE at 1-time GDP per capita	0.55
Thailand 2019^40^	Societal	Lifetime	3%	CE at 1-time GDP per capita	0.76
Colombia-12013^42^	Healthcare payer	Lifetime	5%	Not CE	0.55
Iran 2018^43^	Healthcare system	20 years	3%	CE at 3-time GDP per capita	0.59
UK 2020^41^	NHS payer perspective	Lifetime	3.5%	CE at 1-time GDP per capita	0.64
Latin America 2015^44^	Healthcare system	Lifetime	5%		
Argentina				Not CE	0.64
Bolivia				Not CE	0.66
Brazil				Not CE	0.64
Chile				4.3% probability of being CE at 3-time GDP per capita	0.64
Colombia-2				Not CE	0.66
Peru				Not CE	0.68
Uruguay				26.6% probability of being CE at 3-time GDP per capita	0.64
Indonesia 2022^45^	Societal	Lifetime	3%	Not CE	*
Indian reference study 2019	Societal	Lifetime	3%	Not CE at 1-time GDP per capita	0.76†

* Did not pass through two critical objective criteria and hence, was knocked out.

† Assessment done using general transferability index only.

CE, cost-effective; GDP, gross domestic product; NHS, National Health Service.

Based on original conclusion of the included studies, it was observed that annual trastuzumab therapy was cost-effective at one-time GDP per-capita threshold in four countries namely Thailand, Netherlands, Cyprus and UK.^3639^,^41^ Two studies found trastuzumab to be cost-effective at three-time GDP per-capita.^37 38^ There was a 4.3% and 26.6% probability for trastuzumab to be cost-effective at three-time GDP per-capita in Chile and Uruguay, respectively.^44^ The remaining three studies and evaluation for Argentina, Colombia and Peru (multicentric Latin-American study) found trastuzumab to be cost-ineffective even at a threshold of three-time GDP per-capita.^3542^,^44^ The Indian reference study concluded the use of annual trastuzumab to be cost-ineffective.^30^ Details of the results from the selected EEs as originally reported are mentioned in online supplemental appendix 6a.

#### Intensity-modulated radiation therapy

For IMRT, a total of three studies were identified, which were conducted for Canada, USA and Brazil, respectively^46^,^48^ (table 2). The studies were undertaken either from a healthcare payer or healthcare system perspective using a lifetime horizon. Discounting was done at a rate of 5% and 3% for Canada and US study, respectively, while the rate of discounting was not reported by the Brazilian study. The findings from these selected studies concluded that IMRT was a cost-effective intervention in comparison to 3D-CRT. The Brazilian study was excluded from the adaptation exercise as its global IT (0.46) was below the threshold value of 0.5. The Indian reference study for IMRT undertook the analysis from a societal perspective, using a lifetime horizon and discounting at a common rate of 3% and concluded IMRT to be a cost-ineffective intervention.^33^ Details of the results from the selected EEs as originally reported are mentioned in online supplemental appendix 6b.

**Table 2:** Summary of included studies for IMRT

Country	Perspective	Time horizon	Discount rate	Conclusion	Global transferability index
Canada 2012^46^	Healthcare Payer	Lifetime	5%	CE at 1-time GDP per capita	0.64
USA 2013^47^	Healthcare Payer	2 years and lifetime	3%	CE at 1-time GDP per capita	0.69
Brazil 2018^48^	Healthcare system	Lifetime	Not reported	CE at 1-time GDP per capita	0.46
Indian reference study 2020	Societal	Lifetime	3%	Not CE at 1-time GDP per capita	0.73*

* Assessment done using general transferability index only.

CE, cost-effective; GDP, gross domestic product; IMRT, intensity-modulated radiation therapy.

### Cost and outcome adaptation

#### Scenario I: trastuzumab

**Figure 5:**
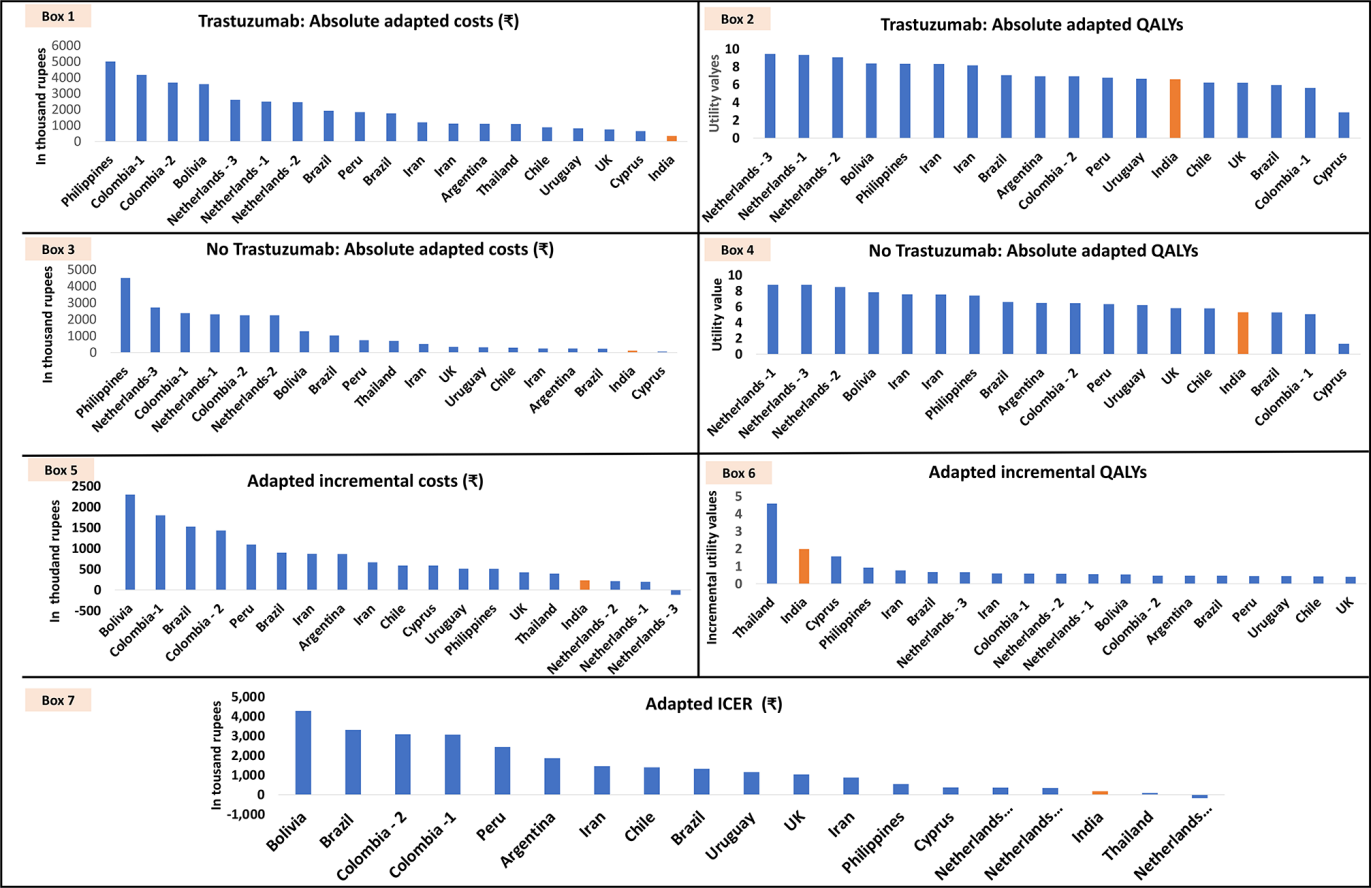
Adapted outcomes (using scenario I for cost adaptation) from economic evaluations on trastuzumab. ICER, incremental cost-effectiveness ratio; QALY, quality-adjusted life-year.

As compared with the Indian reference study, the adapted valuation of absolute cost (as calculated using scenario I) from most of the selected EEs (except the Cyprus study) was 2–40 times higher for the comparator-arm and 2–15 times higher for the intervention-arm (boxes 1 and 3 of figure 5; online supplemental appendix 7a). The adapted values of QALY for both the comparator (0.25–1.65 times) and intervention arms (0.41–1.35 times), were comparable to the QALY per-person estimated in the Indian reference study (boxes 2 and 4 of figure 5; online supplemental appendix 7a). Similar to the absolute cost values, the adapted values of incremental cost ranged from 0.83 to 10 times (except the scenario analysis from the Netherland’s study, which showed trastuzumab to be cost-saving), while incremental QALYs ranged from 0.21 to 2.3 times as compared with the findings of the Indian reference study (boxes 5 and 6 of figure 5; online supplemental appendix 7a). After adaptation (scenario I), the ICERs were significantly higher for most of the studies (except the Thailand study and a scenario analysis from Netherland’s study) by 2–24 times as compared with the findings in the Indian reference study. In conclusion, the adapted ICERs (except the EE from Thailand and scenario analysis of Netherland’s study) showed trastuzumab to be cost-ineffective for India (box 7 of figure 5; online supplemental appendix 7a).

#### Scenario I: IMRT

**Figure 6:**
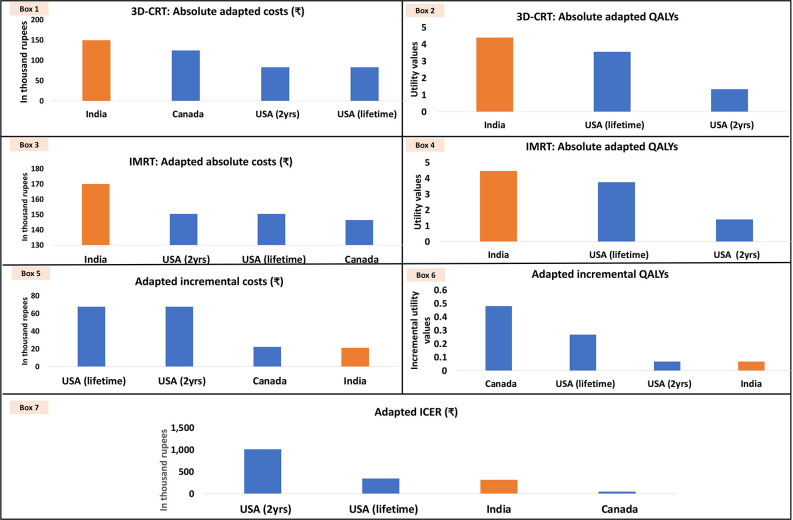
Adapted outcomes (using scenario I for cost adaptation) from economic evaluations on IMRT. 3D-CRT, 3-dimensional conformal radiation therapy; ICER, incremental cost-effectiveness ratio; IMRT, intensity-modulated radiation therapy; QALY, quality-adjusted life-year.

The adapted values of both absolute cost and QALYs estimates for both the treatment arms were on a lower side as compared with the findings of the Indian reference study (boxes 1–4 of figure 6; online supplemental appendix 7b). On the contrary, the adapted incremental cost and QALYs were higher as compared with the values estimated in the Indian reference study (boxes 5 and 6 of figure 6; online supplemental appendix 7b). In terms of adapted ICERs, while findings from the US study showed IMRT as cost-ineffective, the Canadian EE concluded IMRT as cost-effective for India (box 7 of figure 6; online supplemental appendix 7b).

#### Scenario II

With regard to trastuzumab, the adapted cost values in the comparator varied by 0.03 times on the lower side to 23 times on the higher side of the results in the Indian reference study. Similarly, for the intervention arm the adapted cost varied by 0.23 times on the lower side to 8 times on the higher side of the values in the Indian reference study (online supplemental appendix 8a). A majority (69%) of the adapted incremental cost values were lower than the findings of the Indian reference study and ranged from 0.01647 to 0.9 times (online supplemental appendix 8a) of the values reported in Indian reference study. Like the incremental costs, the adapted ICERs were also lower for a majority (62%) of the studies and concluded trastuzumab to be cost-effective for India (online supplemental appendix 8a).

In case of IMRT, the adapted values of absolute costs for both the treatment arms were significantly lower (0.010–0.028 times) as compared with the findings of the Indian reference study (online supplemental appendix 8b). In terms of adapted ICERs, the findings from the selected EEs showed IMRT as cost-effective for India.

### Price-benchmarking

**Figure 7:**
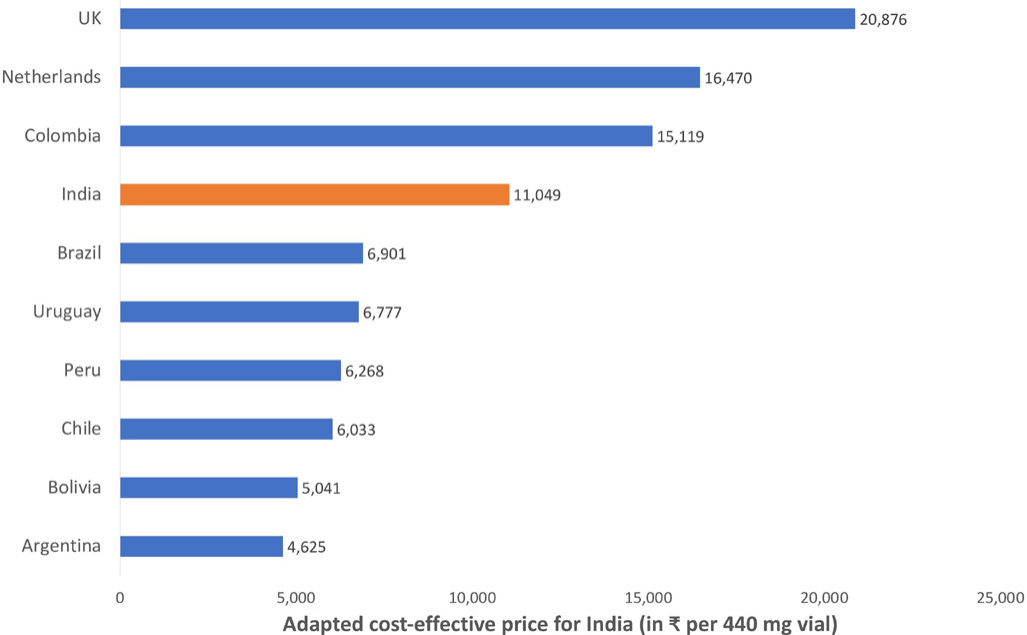
Price-benchmarking for trastuzumab.

The adapted price at which trastuzumab was cost-effective in India was calculated for nine countries, and ranged from ₹4625 to ₹20876 as shown in figure 7. When compared with the cost-effective price for India (as estimated in the Indian reference study), the adapted prices were not comparable in absolute terms and varied from half to twice of the drug price reported in the Indian reference study.

### Subgroup analysis

#### Trastuzumab: scenario 1

The adapted ICERs of the studies from high-income countries were found to be 4.5 times (range: 2–8 times) higher than the ICERs reported in the Indian reference study (online supplemental appendix 7a) except the Dutch study, which found trastuzumab to be cost saving. Similarly, the adapted ICERs from middle-income countries were found to be 11. 5 times (range: 0.48–24 times) higher than the Indian reference study’s results. The adapted ICERs from studies that had assumed a societal perspective were around 2 times (range: 0.48–3.05 times) higher than the values in the Indian reference study, while the adapted ICER from studies that had assumed a health system perspective were 9 times higher (range: 2–24 times) than the Indian reference study results. The adapted ICERs from studies that had used HERA trial as the source of effectiveness data, were 2–24 times higher than the Indian reference study results. The Dutch study was found to be an exception in this case as well, as it found trastuzumab to be cost saving. The adapted ICERs from studies that had used a discount rate similar to the reference study, that is, of 3% for adjusting future costs and consequences were found to be 2.5 times (range: 0.5–5 times) higher than the ICER reported in the Indian reference study.

#### Trastuzumab: scenario II

The adapted ICERs of the studies from high-income countries were found to be 0.25 times lower (range: 0.04–0.58 times) than the ICERs reported in the Indian reference study (online supplemental appendix 8a). However, the adapted ICERs from middle-income countries were 2.5 times (range: 0.15–13 times) higher than the Indian reference study’s results. While the adapted ICERs of studies using a societal perspective were 12% lower than the Indian reference study, those using a health system perspective reported an ICER which after adaptation was 2 times (0.14–13 times) higher than the Indian reference study. The studies that had used HERA trial as the source of effectiveness data found that the ICER after adaptation varied from 0.04 times on a lower side to 12 times higher as compared with Indian reference study’s findings, an exception to the above was the finding from the Netherland’s study whose ICER showed the use of trastuzumab to be cost saving. Lastly, the adapted ICERs from studies that had used a discount rate of 3% for adjusting future costs and consequences were found to be 0.25 times (range: 0.13–0.5 times) lower than the ICER reported in the Indian reference study.

#### Price-benchmarking

There was a significant heterogeneity in findings on the benchmark cost-effective price from studies done elsewhere. While the adjusted price estimated from half of the studies from high incomes countries was on the lower side (0.55–0.61 times) as compared with the Indian reference study, it was found to be on the higher side (1.5–1.9 times) based on adaptation from other studies. Using the studies from middle-income countries the value of adapted benchmark price was on the lower side (0.4–0.63) compared with the Indian reference study, except the Columbian study that estimated the benchmark cost-effective price value to be 1.3 times higher as compared with the price in the Indian reference study.

## Discussion

There has been a lot of debate on how to ‘generalise’ or ‘translate’ the findings of an EE to other countries’ contexts, given that there is usually a strong time constraint to make policy decisions.^11 34^ Over the years, there has been a lot of work around the development of different checklists, summarising the list of various factors that could potentially cause or lead to uncertainties while generalising, transferring, or adapting the results of an EE to other settings.^34^ Broadly these factors are summarised under three specific categories of methodological features (perspective, discount rate, approaches for direct and indirect cost assessment), healthcare system factors and population characteristics.^21 34^ Many of these factors, either alone or in interaction with other factors, affect an EE’s cost and valuation of outcomes, thereby influencing the ICER calculation.

### Summary and interpretation of the findings

The present analysis is the first of its kind to use selected aHTA approaches for EE (including literature review, quality and transferability appraisal, and costs, outcome and price adjustments), and to assess it against the traditional full EE for measuring the validity and accuracy of the findings. In terms of direction and magnitude, the adapted cost estimates varied considerably, while the adapted QALYs did not differ much, when matched with the results of the Indian reference study.

The adapted ICERs from around 90% of the selected EEs on trastuzumab were higher from the threshold value of one-time GDP per-capita of India and showed trastuzumab to be cost-ineffective for India, which matches with the overall conclusion of the Indian reference study. However, these adapted ICERs were on an average about eight times higher than the threshold value for India. Whereas the ICER reported in the Indian reference study was only 23% higher than the threshold value. This huge difference between the adapted ICER and the threshold value shows a higher level of uncertainty and needs to be interpreted with caution.

In a hypothetical scenario with unlimited resources where any intervention found to be cost-effective will be funded as a part of HBP, use of estimates from aHTA may be acceptable. However, decisions in most LMICs are usually made on the margins in the context of limited resources or finite budget, which involves prioritisation. This implies that even if several interventions are found to be cost-effective, only a few of them may become a part of the existing HBP in a particular year, which will be based on the relative ranking of ICERs. So, having a dichotomous broad conclusion that whether a particular intervention is cost-effective or not, is insufficient for LMICs, where decisions are made in the context of limited healthcare resources. The adapted ICERs need to be in a close range of the original ICERs (of the full EE), and the relative rankings should also be in the same order (as from full HTA). So, from a broader point of view of resource prioritisation, aHTA findings may result in inappropriate allocation of resources.

Moreover, the absolute difference of ICERs generated from aHTA also need appropriate consideration. In situations where a full EE concludes an intervention to be cost-effective, with an ICER marginally (10%–20%) below the threshold, an aHTA for the same intervention could contradict the findings of a full EE and conclude this intervention to be cost-ineffective, given the variation in the ICERs generated from a full EE and aHTA, respectively. Therefore, in cases where the ICER is in close range of the threshold, use of aHTA increases uncertainty. Whereas, in cases where ICERs are on the extremes, that is either significantly below the cost-effectiveness threshold or more than 2–3 times of the threshold value, then the conclusion drawn using the aHTA findings are more likely to be in line with full EE.

In some situations, the findings from the aHTA may lead to inconclusive evidence. For example, in the case of IMRT, half of the adapted ICERs shows IMRT to be cost-effective and remaining half shows it to be cost-ineffective. In such situations, the only rationale is to undertake a context-specific full HTA, to assess the true cost-effectiveness of the intervention.

A direct adaptation of model by changing the parameters that potentially differ between the study country (for which EE was actually conducted) and the decision country (for which adaptation is being done) could address most of the factors causing uncertainties in transferability.^17 49^ However, most of the time, researchers do not have access to original model used in EE. Furthermore, even if the model is accessible, comprehending the intricate details and calculations that went through the complex model structure again becomes difficult and equally time-consuming as developing a new model and conducting a full HTA.

It was presumed that the adapted findings from EEs of countries with similar socioeconomic or population characteristics to India might show results which are closer to the Indian reference study. However, subgroup analysis showed that both the adapted ICER values (either in scenario I or II) as well as the benchmark cost-effective price from the selected EEs of trastuzumab, irrespective of the similarity in contextual factors, differed from the findings of the Indian reference study. Likewise, the adapted ICERs from studies that had used a similar source of effectiveness data, or discount rate as used in the Indian reference study, also did not show any specific similarity in absolute terms or direction with the findings of the Indian reference study. However, when the perspective of analysis was the same there was considerably lesser variation in the adapted and Indian reference study estimates.

Specifically, for scenario II (ie, using each of the three correction factors for cost adaptation), it was observed that the conclusion from around one-third of the selected EEs showed trastuzumab as cost-effective (online supplemental appendix 8a). Furthermore, the adapted ICERs, especially from the high-income countries’ studies, dropped down significantly by 75%, which does not match with or reflect the actual scenario. All these findings need to be interpreted with due caution. Over-adjustment of cost estimates with the use of both correction factors A and B, could be one of the reasons leading to a drastic decrease in the adapted values. Moreover, this effect was more pronounced in case of high-income countries, probably due to the fact that there is a huge difference in the GDP and health expenditure of these countries as compared with India, which is also reflected in the values of correction factor of A and B for these countries.

As estimated using price-benchmarking analysis, the adapted threshold cost-effective price for trastuzumab varied appreciably from 37% on lower-side to 89% on higher-side, when compared with the cost-effective price of ₹11 049 estimated in the Indian reference study. Although the average adapted price (and even the individual adapted price from most of the selected EEs) was on the lower side of the price estimated in the Indian reference study, large variation among the individual adapted estimates, creates uncertainties and ambiguity in ascertaining a price value at which a particular drug might be cost-effective locally. Nevertheless, evidence from price-benchmarking provides a strong imperative for price regulations and negotiations in situations where the current price is significantly above the prices estimated using the price-benchmarking analysis. However, price-benchmarking can only be used for adapting the price of pharmaceutical interventions and it is not possible to use this approach for device (or healthcare programme), because of the range of factors that goes in estimating the cost per patient in case of medical devices or programme.

A fundamental conclusion from the present analysis is that cost is difficult to adapt and hard to generalise, whereas the health outcomes are closer across settings. While the existing cost correction factors adjusted for the differences in the quantity and prices of the healthcare resources, however, the variation in other important factors such as skill mix of personnel delivering care, clinical practice variability, the level at which healthcare is delivered, extent of technical efficiency across countries were still unaccounted, which might have led to uncertainty in the adapted cost estimates. Moreover, elementary differences in the context-specific assumptions, while designing an EE and its model structure, are difficult to adjust and might lead to dissimilarities in the resource use and the associated costs. It is recommended that in future, researchers should focus on developing more refined and robust methods for predicting costs across settings.

In view of the findings from our analysis, which concluded that adapted QALYs showed lesser variation when compared with the Indian reference studies, we consider that the QALY adjustment factor is more accurate than cost correction factors and could potentially be used in future for adjusting population characteristics across settings. Furthermore, for adjusting QALYs a simpler and more direct approach could be to use the average ratio of tariff values of corresponding health states in the value set of the study country and the decision country. However, while we adjusted for the difference in the age of onset of disease, life expectancy and health status preferences, which are fundamental to the calculation of QALYs, we also recognise that there could be other factors related to source of treatment effectiveness, compliance, adherence, etc. that could potentially create uncertainty in the estimation of adapted QALYs across settings. Considering this, it is advised undertaking future research to further improve aHTA methods for the adjustment of health outcomes.

Nevertheless, our study has a few limitations. First, literature review was conducted in one database only which might have resulted in some studies being missed. However, the objective of the study was to assess the adaptability of existing EEs and comparing the adapted results to those of a full EE, and not to identify all existing EEs published for a given topic. Second, we undertook adaptation for one drug and one technology; therefore, our results might not be generalisable to other HTA types including programmatic evaluations. However, given the significant heterogeneity in the healthcare structures, context and programme delivery, it is likely that there will be greater uncertainty in applying aHTA to EE of healthcare programmes. Third, for adapting cost estimates we used national level GDP or health care expenditure estimates which may have over or underestimated the costs, especially in case of studies undertaken in HICs such as Canada and the USA where there are significant variations in the price of resources across regions. While the use of subnational or regional estimates for cost adaptation may have produced more accurate estimates, however, we believe this would not change the overall conclusion of our study findings, as the overall objective of our study was to identify whether or not the adapted findings are generalisable to Indian context. Fourth, disaggregated data on QALYs was not reported in two of the studies, and hence absolute QALYs were not adapted. However, it is believed this would not impact our overall conclusions because the adapted QALYs were relatively closer to the QALYs reported in the Indian reference study and had minimal impact on the adapted ICERs. Fifth, the conclusions for aHTA for medical devices were based only on two studies. It is recommended to further carry out more validation in the context of medical devices by adapting findings from a larger number of diverse EEs. Finally, we have assessed only the accuracy of the adapted findings for EE, by comparing the adapted results with those reported in the Indian reference study. However, it is recommended that in addition to accuracy, researchers should also try to evaluate the appropriateness and robustness of the aHTA methods including those beyond EE, in future evaluations.

## Conclusion

Despite the limitations of aHTA approaches, it still offers some relevant evidence as compared to no evidence at all. However, these approaches still cannot be considered as a replacement of the traditional HTA methods, even for decisions that require immediate evidence. aHTA as an approach may be considered as one of the tools in the overall traditional HTA process. First, aHTA may be used for summarising the existing evidence on the clinical efficacy, safety, cost and cost-effectiveness from the globally published EE or HTA reports. Second, aHTA can be used for ‘topic prioritisation’ in the schematic flow of HTA process, whereby those interventions with an adapted ICERs beyond twice or thrice of threshold values, that is, interventions that are highly cost-ineffective, can directly be ruled out. This will save time and resources from conducting a full HTA for these interventions and the efforts can be directed towards other technologies which are not well studied internationally or where HTA evidence is inconclusive.

Overall, the present analysis concludes that the adjusted findings from the aHTA approaches for EE should be interpreted with caution, even if the adapted results are generated using HTA evidence from a nation with similar characteristics to the decision country. Further, given the increased relevance of transferability of HTA evidence to other countries’ contexts, there is a need to develop more robust aHTA approaches for cost adjustment.

## Supplementary material

10.1136/bmjgh-2023-012277 online supplemental file 1

## Data Availability

All data relevant to the study are included in the article or uploaded as online supplemental information.
