# Supplementary material for: Validating the rigour of adaptive methods of economic evaluation
Source: BMJ Glob Health. 2023 Sep 26;8(9):e012277. doi: 10.1136/bmjgh-2023-012277 (PMC10533726; doi:10.1136/bmjgh-2023-012277)
Supplement: online supplemental file 1 [file bmjgh-2023-012277supp001.pdf]

Appendices

Appendix 1: PubMed search strategy for transtuzumab

#1 breast neoplasm
#2 breast carcinoma
#3 cancer of breast
#4 mammary cancer
#5 (breast neoplasm[MeSH]
#6 **1 OR #2 OR #3 OR #4 OR #5**
#7 trastuzumab
#8 herceptin
#9 Epidermal growth factor receptor 2 OR
#10 her2
#11 erbB-2 receptor
#12 **#7 OR #8 OR #9 OR #10 OR #11**
#13 cost benefit analys*
#14 cost-benefit analysis[MeSH]
#15 cost utility analys*
#16 cost-utility analysis[MeSH]
#17 cost effectiveness
#18 cost effectiveness analys*
#19 cost-effectiveness analysis[MeSH]
#20 cost minimization analys*
#21 cost-minimization analysis[MeSH]
#22 economic evaluation*
#23 pharmaco-economic evaluation
#24 pharmaco-economic analys*
#25 pharmaco-economic analysis[MeSH]
#26 health technology assessment*
#27 **#13 OR #14 OR #15 OR #16 OR #17 OR #18 OR #19 OR #20 OR#21 OR #22 OR #23 OR #24 OR #25 OR #26**
#28 **#6 AND #12 AND#27**
Filters: Publication date: Last 10 years to 31.5.2023
No language restrictions

Appendix 2: PubMed search strategy for IMRT

#1 Head and Neck Neoplasms [MeSH]
#2 Neoplasms, Head and Neck
#3 Head, Neck Neoplasms
#4 Cancer of Head and Neck
#5 Head and Neck Cancer
#6 (Cancer of the Head and Neck
#7 Head and neck squamous cell carcinoma
#8 Oropharyngeal Neoplasms [MeSH]
#9 Neoplasm, Oropharyngeal
#10 Cancer of Oropharnyx
#11 Oropharnyx Cancer
#12 Oropharyngeal Cancer
#13 Laryngeal Neoplasms [MeSH)
#14 Larynx Neoplasms
#15 Cancer of Larynx
#16 Laryngeal Cancer
#17 Larynx Cancer
#18 Supraglottic Cancer
#19 Cancer of supraglottis
#20 Cancer of supraglottic region
#21 Nasopharyngeal Neoplasms [MeSH]
#22 Cancer of Nasopharynx
#23 Nasopharynx Cancers
#24 Nasopharyngeal Cancer
#25 Hypopharyngeal Neoplasms [MeSH]
#26 Hypopharyngeal Cancer
#27 Cancer, Hypopharyngeal
#28 Hypopharynx cancer
#29 Cancer of hypopharynx
#30 Mouth Neoplasms [MeSH]
#31 Oral Cancer)
#32 Paranasal Sinus Neoplasms[MeSH]
#33 Paranasal Sinus Cancer
#34 Cancer, Paranasal Sinus
#35 Salivary Gland Neoplasms [MeSH]
#36 Cancer of Salivary Gland
#37 Salivary Gland Cancer
#38 Lip Neoplasms [MeSH])
#39 Cancer of Lip
#40 Lip Cancers
#41 Tongue Neoplasms [MeSH]
#42 Cancer of Tongue
#43 Tongue Cancers
#44 Tongue Neoplasm
#45 Sinonasal cancer

#46 **#1 OR #2 OR #3 OR #4 OR #5 OR #6 OR #7 OR #8 OR#9 OR #10 OR #11 OR #12 OR #13 OR #14 OR #15 OR #16 OR #17 OR #18 OR #19 OR #20 OR#21 OR #22 OR #23 OR #24 OR #25 OR #26 OR #27 OR #28 OR #29 OR #30 OR #31 OR #32 OR #33 OR #34 OR #35 OR #36 OR #37 OR #38 OR #39 OR #40 OR #41 OR #42 OR #43 OR #44 OR #45**
#47 Radiotherapy, Intensity-Modulated"[MeSH]
#48 Intensity-Modulated Radiotherapies
#49 Radiotherapy, Intensity Modulated
#50 Intensity-Modulated Radiation therapy
#51 IMRT
#52 **#47 OR #47 OR #48 OR #49 OR #51**
#53 Conventional radiation therapy
#54 Conventional radiotherapy Cobalt radiotherapy
#55 Cobalt radiation therapy
#56 2 Dimensional radiotherapy
#57 2 Dimensional Radiation therapy
#58 2 DRT
#59 2drt
#60 2-DRT
#61 3 DCRT
#62 3 Dimensional conformal radiotherapy
#63 3 Dimensional conformal radiation therapy
#64 3 dcrt
#65 Linear accelerator
#66 **#52 OR#53 OR #54 OR #55 OR #56 OR #57 OR #58 OR #59 OR #60 OR#61 OR #62 OR #63**
#67 cost benefit analys*
#68 cost-benefit analysis[MeSH]
#69 cost utility analys*
#70 cost-utility analysis[MeSH]
#71 cost effectiveness
#72 cost effectiveness analys*
#73 cost-effectiveness analysis[MeSH]
#74 cost minimization analys*
#75 cost-minimization analysis[MeSH]
#76 economic evaluation*
#77 pharmaco-economic evaluation
#78 pharmaco-economic analys*
#79 pharmaco-economic analysis[MeSH]
#80 health technology assessment*
#81 **#66 OR #67 OR #68 OR #69 OR #70 OR #71 OR #72 OR #73 OR #74 OR #75 OR #76 OR #77 OR #78 OR #79**
#82 **#46 AND #52 AND #66 AND #81**
#83 Filters: Publication date: Last 10 years to 31.5.202
No language restrictions

Appendix 3: Antonanza’s checklist

GENERAL TRANSFERABILITY INDEX (IT1)	
**Critical objective factors**	**Non-critical objective factors**
1. The relevant parameters needed to calculate the ratio cost/effectiveness are given in the study<br>2. Objectives are presented in a clear, specific and measurable manner<br>3. The variable estimates used in the analysis come from the best available source<br>4. The measurement of costs is appropriate and the methodology for estimation of quantity and unit costs is clearly described<br>5. Health outcome measures are based on valid and reliable scales. Otherwise, the scales used must be fully justified<br>6. The economic model, study methods and components of the costs and effectiveness are presented in a clear manner<br>7. The conclusions and recommendations of the study are justified and based on the study results.	*1. Is the intervention described in sufficient detail?<br>2. Is the comparator described in sufficient detail?<br>3. Is the country in which the economic study took place clearly specified?<br>4. Did the authors correctly state the perspective for the economic analysis?<br>5. Is the target population of the health technology clearly stated or can it be inferred by reading the article?<br>6. Does the article provide sufficient detail about the study sample?<br>7. Have the principal estimates of effectiveness measures been reported?<br>8. Are the results of a statistical analysis of the effectiveness results provided?<br>9. Is the level of reporting of benefit data adequate (incremental analysis, statistical analyses)?<br>10. Are the cost components used in the analysis presented?<br>11. Are unit price for resources given?<br>12. Are costs and quantities reported separately?<br>13. Is the price year given?<br>14. Is the currency unit reported?<br>15. Are quantitative &/or descriptive analyses conducted to explore variability from place to place<br>16. Did the authors discuss the generalizability of results?*
**SPECIFIC TRANSFERABILITY INDEX (IT2)**	
**Critical subjective factors**	**Non-critical subjective factors**
1. The evaluated technology is used in the new health context.<br>2. The comparator is available or used in the new context.<br>3. Treatment and comparator data, as well as relevant epidemiological parameters for the technology, are valid in the new context.<br>4. The study perspective coincides with that used in the new context	*1. Cost components correspond to the medical practice related to the evaluated technology in the original study. If medical practice differs in new context, additional costs components must be taken into account.<br>2. The model connecting variables and parameters can be adapted to the new context.<br>3. Life expectancy is similar in both contexts.<br>4. Health-status preferences are similar in both contexts. (Applicable to cost/utility analyses)<br>5. Productivity measures are similar in both contexts<br>6. The evolution of the disease is similar in both contexts.<br>7. The applied discount rate is similar in both contexts.<br>8. Costs and health effects data are presented in current and discounted units*

Appendix 4: Calculation of cost correction factors

**Name of country (Year)**	**Country**		**India**		**Correction factor (A)**	**Correction factor (B)**
	**Health care expenditure per capita (PPP adjusted) in US dollars**	**GDP per capita (PPP adjusted) in US dollars**	**Health care expenditure per capita (PPP adjusted) in US dollars**	**GDP per capita (PPP adjusted) in US dollars**		
Iran (2018)	1225.0	14535.9	181.5	6182.9	**0.121**	**0.244**
Iran (2014)	1164.0	17388.9	141.4	4236.7	**0.121**	**0.244**
Brazil (2022)	1438.7	15020.4	195.6	6675.4	**0.135**	**0.444**
Philippines	321.6	8121.0	181.5	6183.0	**0.564**	**0.761**
Colombia 2013	751.3	10732.0	141.4	4237.0	**0.188**	**0.395**
Thailand	523.0	14871.0	161.8	4861.2	**0.309**	**0.326**
Netherlands	4989.0	47272.1	161.9	4861.2	**0.032**	**0.103**
Cyprus	2532.9	38288.0	181.5	6182.9	**0.072**	**0.161**
Canada	4153.8	38865.4	138.7	3912.4	**0.033**	**0.100**
USA	8342.6	51602.9	161.8	4861.2	**0.019**	**0.094**
UK	3997.3	40218.7	189.6	5057.2	**0.047**	**0.125**
Peru	511.0	10767.0	161.8	4861.0	**0.316**	**0.451**
Colombia (2015)	802.7	12018	161.8	4861.0	**0.201**	**0.404**
Bolivia	311.1	5921.0	161.8	4861.0	**0.520**	**0.820**
Argentina	1929.2	19641.0	161.8	4861.0	**0.083**	**0.2475**
Brazil (2015)	1164.0	15046.0	161.8	4861.0	**0.139**	**0.323**
Chile	1510.0	21512.0	161.8	4861.0	**0.107**	**0.225**
Uruguay	1594.4	18192.0	161.8	4861.0	**0.101**	**0.267**

Appendix 5: Calculation of quality adjusted life years (QALY) correction factors

**Name of country**	**Life Expectancy at birth (in years)**	**Median age of disease onset (in years)**	**Correction factor (D)**	**Correction factor (E)**
India	70	**50**	-	-
Iran_2014	77	50	0.740	0.920
Iran_2018	77	45	0.625	1.015
Brazil_2022	76	49	0.740	1.10
Philippines	71	50	0.95	0.975
Columbia_2013	77	50	0.740	1.00
Thailand	77	50	0.740	0.935
Netherlands	82	51	0.645	1.039
Cyprus	81	52.3	0.70	1.256
UK	81	55	0.77	1.278
Peru	77	55	0.90	0.8786
Colombia_2015	77	55	0.90	0.8786
Bolivia	72	55	1.17	0.8786
Argentina	77	55	0.91	0.8786
Brazil_2015	76	55	0.95	0.8786
Chile	80	55	0.80	0.8786
Uruguay	78	55	0.87	0.8786

Appendix 6a: Results of economic evaluations originally reported by the authors: Trastuzumab

Year	Country	Reported currency	Comparator		Intervention		Incremental outcomes		ICER (₹)
			Cost (₹)	QALYs	Cost (₹)	QALYSs	Cost (₹)	QALYSs	
2017	Netherlands[1]	EURO	15,401,278	13.103	16,689,481	13.930	12,88,203	0.827	1,557,682
2017	Netherlands[2]	EURO	15,024,211	12.666	16,445,263	13.527	14,21,052	0.861	1,650,466
2017	Netherlands[3]	EURO	18,192,259	13.104	17,406,560	14.098	-7,85,699	0.994	-790,441
2020	Cyprus	EURO	292,555	1.51	3,478,754	3.3	31,86,199	1.79	1,779,999
2015	Uruguay	US$	795,660	8.15	2,114,640	8.73	13,18,980	0.58	2,274,103
2015	Chile	US$	886,440	8.26	2,680,680	8.86	17,94,240	0.6	2,990,400
2020	UK	GBP	1,882,834	8.6	4,292,902	9.2	24,10,068	0.6	4,016,780
2018	Philippines	PHP	5,173,259	7.99	5,756,505	8.99	5,83,246	1	583,245
2013	Colombia	US$	3,441,895	6.83	6,048,897	7.62	26,07,002	0.79	3,300,002
2014	Iran	US$	566,131	11.11	2,604,168	11.98	20,38,037	0.87	2,342,571
2019	Thailand*	US$	1474267	-	2305171	-	830904	4.59	181025
2015	Peru	US$	1,121,400	7.93	2,787,480	8.49	16,66,080	0.56	2,975,142
2015	Colombia	US$	3,823,440	8.1	6,258,480	8.68	24,35,040	0.58	4,198,344
2015	Bolivia	US$	1,073,340	7.59	3,001,080	8.11	19,27,740	0.52	3,707,192
2015	Argentina	US$	651,480	8.12	3,054,480	8.7	24,03,000	0.58	4,143,103
2015	Brazil	US$	485,940	7.88	3,732,660	8.43	32,46,720	0.55	5,903,127
2022	Brazil	US$	1,320,547.	6.48	2,475,647	7.31	11,55,100	0.83	1,391,686
2018	Iran	EURO	1,069,345	11.1	2,438,586	12.22	13,69,241	1.12	199,556

[1]Real-world scenario; [2]Guideline scenario; [3]Trial scenario; *Absolute number of QALYs not reported by the authors of the original study

Appendix 5b: Results of economic evaluations originally reported by the authors: IMRT

Year	Country	Reported currency	Comparator		Intervention		Incremental outcomes		ICER (₹)
			Cost (₹)	QALYs	Cost(₹)	QALYSs	Cost (₹)	QALYSs	
2013	US (2yrs)	US$	605,342	1.817	1,100,360	1.909	4,95,018	0.092	5,380,630
2013	US (lifetime)	US$	605,335	4.855	1,100,361	5.123	4,95,026	0.268	1,847,109
20012	Canada*	CAD	660,196	-	778,667	-	1,18,471	0.480	246,814

*Absolute number of QALYs not reported by the authors

Appendix 7a: Results of cost adaptation using Scenario I correction factor B and C: Trastuzumab

Year	Country	Comparator		Intervention		Incremental outcomes		ICER (₹)
		Cost (₹)	QALYs	Cost (₹)	QALYSs	Cost (₹)	QALYSs	
2017	Netherlands[@1]	2,298,068	13.10	2,490,284	13.93	192,216	0.83	232,425
2017	Netherlands[@2]	2,241,804	12.67	2,453,843	13.53	212,039	0.86	246,270
2017	Netherlands[@3]	2,714,518	13.10	2,597,281	14.09	-117,236	0.99	-117,944
2020	Cyprus[@]	53,934	1.51	641,321	3.30	587,387	1.79	328,149
2015	Uruguay[@]	308,497	8.15	819,898	8.73	511,401	0.58	881,725
2015	Chile[@]	290,656	8.26	878,972	8.86	588,316	0.60	980,526
2020	UK[@]	330,315	8.60	753,125	9.20	422,810	0.60	704,683
2019	Philippines[#]	4,496,496	7.99	5,003,441	8.99	506,945	1.00	506,945
2013	Colombia[#]	2,369,763	6.83	4,164,698	7.62	1,794,935	0.79	2,272,070
2014	Iran[#]	240,556	11.11	1,106,544	11.98	865,988	0.87	995,388
2019	Thailand[#*]	699,273	-	1,093,387	-	394,114	4.59	85,863
2015	Peru[#]	734,596	7.93	1,825,996	8.49	1,091,400	0.56	1,948,929
2015	Colombia[#]	2,243,922	8.10	3,673,011	8.68	1,429,090	0.58	2,463,948
2015	Bolivia#	1,278,525	7.59	3,574,781	8.11	2,296,256	0.52	4,415,877
2015	Argentina[#]	233,959	8.12	1,096,922	8.70	862,963	0.58	1,487,867
2015	Brazil[#]	227,805	7.88	1,749,845	8.43	1,522,040	0.55	2,767,345
2022	Brazil[#]	1,023,514	6.48	1,918,795	7.31	895,281	0.83	1,078,652
2018	Iran[#]	519,266	11.10	1,184,160	12.22	664,894	1.12	593,655

[@]High income countries; [#]middle-income countries; [1]Real-world scenario; [2]Guideline scenario; [3]Trial scenario; *Absolute number of QALYs not reported by the authors of the original study

Appendix 7b: Results of cost adaptation using Scenario I (correction factor B and C): Intensity Modulated Radiotherapy (IMRT)

Year	Country	Comparator		Intervention		Incremental outcomes		ICER (₹)
		Cost (₹)	QALYs	Cost (₹)	QALYSs	Cost (₹)	QALYSs	
2013	US (2yrs)	82,744	1.817	150,408	1.909	67664	0.092	735,478
2013	US (lifetime)	82,744	4.855	150,408	5.123	67665	0.268	252,481
2012	Canada*	124,108	-	146,379	-	22,271	0.48	46,398

*Absolute number of QALYs not reported by the authors of the original study

Appendix 8a: Results of cost adaptation using Scenario II (correction factor A, B, and C): Trastuzumab

Year	Country	Comparator		Intervention		Incremental outcomes		ICER (₹)
		Cost (₹)	QALYs	Cost (₹)	QALYSs	Cost (₹)	QALYSs	
2017	Netherlands@1	74,547	13.10	80,782	13.93	6,235	0.83	7,539
2017	Netherlands@2	72,722	12.67	79,600	13.53	6,878	0.86	7,988
2017	Netherlands@3	88,056	13.10	84,253	14.09	-3,803	0.99	-3825
2020	Cyprus@	3,865	1.51	45,963	3.30	42,098	1.79	23,518
2015	Uruguay@	31,316	8.15	83,229	8.73	51,913	0.58	89,505
2015	Chile@	31,153	8.26	94,211	8.86	63,057	0.60	105,095
2020	UK@	15,669	8.60	35,726	9.20	20,057	0.60	33,428
2019	Philippines#	2,537,930	7.99	2,824,062	8.99	286,132	1.00	286,132
2013	Colombia#	445,843	6.83	783,538	7.62	337,696	0.79	427,463
2014	Iran#	29,212	11.11	134,375	11.98	105,163	0.87	120,877
2019	Thailand#*	216,029	-	337,783	-	121,755	4.59	26,526
2015	Peru#	232,670	7.93	578,351	8.49	345,681	0.56	617,287
2015	Colombia#	452,446	8.10	740,597	8.68	288,150	0.58	496,810
2015	Bolivia#	665,111	7.59	1,859,663	8.11	119,4552	0.52	2,297,215
2015	Argentina#	19,628	8.12	92,027	8.70	723,99	0.58	124,825
2015	Brazil#	31,674	7.88	243,301	8.43	211,627	0.55	384,776
2022	Brazil#	139,125	6.48	260,819	7.31	121694	0.83	146,619
2018	Iran#	76,944	11.10	175,468	12.22	98523	1.12	87,966

@High income countries; # middle-income countries; 1Real-world scenario; 2Guideline scenario; 3Trial scenario; *Absolute number of QALYs not reported by the authors of the original study

Appendix 8b: Results of cost adaptation using Scenario II (correction factor A, B and C): Intensity Modulated Radiotherapy (IMRT)

Year	Country	Comparator		Intervention		Incremental outcomes		ICER (₹)
		Cost (₹)	QALYs	Cost (₹)	QALYSs	Cost (₹)	QALYSs	
2013	US (2yrs)	1605	1.817	2918	1.909	1313	0.092	14,269
2013	US (lifetime)	1605	4.855	2918	5.123	1313	0.268	4,898
2012	Canada*	4145	-	4889	-	744	0.480	1,550

*Absolute number of QALYs not reported by the authors of the original study

Appendix 9a: Results of both cost and QALY adaptation: Trastuzumab

Year	Country	Comparator		Intervention		Incremental outcomes		ICER (₹)
		Cost (₹)	QALYs	Cost (₹)	QALYSs	Cost (₹)	QALYSs	
2017	Netherlands[@1]	2,298,068	8.783	2490284	9.337	192216	0.554	346,960
2017	Netherlands[@2]	2,241,804	8.490	2453843	9.067	212039	0.577	367,485
2017	Netherlands[@3]	2,714,518	8.783	2,597,281	9.450	-117,236	0.666	-176,030
2020	Cyprus[@]	53,934	1.322	641,321	2.888	587,387	1.567	374,848
2015	Uruguay[@]	308497	6.227	819,898	6.670	511,401	0.443	1,154,404
2015	Chile[@]	290656	5.806	878,972	6.228	588,316	0.422	1,394,114
2020	UK[@]	330315	5.812	753,125	6.218	422,810	0.406	1,041,404
2019	Philippines[#]	4,496,496	7.413	50,03,441	8.341	506,945	0.928	546,276
2013	Colombia[#]	2,369,763	5.059	4,164,698	5.644	1,794,935	0.585	3,068,265
2014	Iran[#]	240,556	7.571	1,106,544	8.164	865,988	0.593	1,460,351
2019	Thailand[#*]	699,273	-	1,093,387	-	394,114	4.590	85,863
2015	Peru[#]	734,596	6.334	1,825,996	6.781	1,091,400	0.447	2,441,611
2015	Colombia[#]	2,243,922	6.470	3,673,011	6.933	1,429,090	0.463	3,086,587
2015	Bolivia[#]	1,278,525	7.845	3,574,781	8.383	2,296,256	0.537	427,6082
2015	Argentina[#]	233,959	6.486	1,096,922	6.949	862,963	0.463	1,863,851
2015	Brazil[#]	227,805	6.594	1,749,845	7.054	1,522,040	0.460	3,308,783
2022	Brazil[#]	1,023,514	5.277	1,918,795	5.953	895,281	0.676	1,324,380
2018	Iran[#]	519,266	7.564	1,184,160	8.327	664,894	0.763	871,420

[@]High income countries; [#] middle-income countries; [1]Real-world scenario; [2]Guideline scenario; [3]Trial scenario; *Absolute number of QALYs not reported by the authors of the original study

Appendix 9b: Results of both cost and QALY adaptation: Intensity Modulated Radiotherapy (IMRT)

**Year**	**Country**	**Comparator**		**Intervention**		**Incremental outcomes**		**ICER (₹)**
		**Cost (₹)**	**QALYs**	**Cost (₹)**	**QALYSs**	**Cost (₹)**	**QALYSs**	
2013	US (2yrs)	82,744	1.330	150,408	1.398	67,664	0.067	1,009,911
2013	US (lifetime)	82,743	3.550	150,408	3.750	67,665	0.268	345,229
2012	Canada*	124,108	-	146,379	-	22,271	0.48	46,398

*Absolute number of QALYs not reported by the authors of the original study
